# Supplementary material for: GENNUS: generative approaches for nucleotide sequences enhance mirtron classification
Source: NAR Genom Bioinform. 2025 Jun 20;7(2):lqaf072. doi: 10.1093/nargab/lqaf072 (PMC12204755; doi:10.1093/nargab/lqaf072)
Supplement: lqaf072_Supplemental_File [file lqaf072_supplemental_file.pdf]

GENNUS: Generative Approaches for Nucleotide Sequences Enhance Mirtron Classification

Alisson G. Chiquitto[1*], Liliane S. Oliveira[1],

Pedro H. Bugatti[2], Priscila T. M. Saito[2],

Mark Basham[4], Roberto T. Raittz[3], Alexandre R. Paschoal[1,4*]

[1]Department of Computer Science, Federal University of Technology of Paraná - UTFPR, Cornélio Procópio, Brazil.

[2]Department of Computing, Federal University of São Carlos - UFSCar, São Carlos, Brazil.

[3]Laboratory of Bioinformatics, Professional and Technological Education Sector, Federal University of Paraná - UFPR, Curitiba, Brazil.

[4]The Rosalind Franklin Institute - RFI, Didcot, United Kingdom.

*Corresponding author.

Email: chiquitto@gmail.com, paschoal@utfpr.edu.br

Contents

1 Selected classification tools **3**

2 Datasets used as input **4**

3 Number of datasets in the experiments **6**

4 Number of misclassified samples **7**

4.1 UMAP and PaCMAP analysis of training and test sets 9

5 Details of Experiment I **14**

6 Classification performance on other species (Experiment IV) **16**

7 GAN Components **18**

8 Calculating the improvement of the experimental group **22**

1 Selected classification tools

Supplementary Table S1: Tools used in evaluating DA approaches

Tool	Based on	Params †	Reference
cnnMirtronPred	CNN	69,122	(*1*)
dnnPreMiR_CNN	CNN	60,882	(*2*)
dnnPreMiR_RNN	RNN	122,136	(*2*)
dnnPreMiR_CNN_RNN	CNN+RNN	164,488	(*2*)
HumanPreMiRNA_CNN	CNN	183,874	(*3*)
HumanPreMiRNA_RNN	RNN	253,726	(*3*)
miRNAClassification	CNN	170,274	(*4*)

† Trainable parameters

2 Datasets used as input

Supplementary Table S2: Datasets collected from mirtronDB and miRBase

Dataset	specie	mirtrons	canonical miRNAs	Total
Dataset I	*H. sapiens*	417	707	1,124
Dataset II	*H. sapiens*	165	952	1,117
Dataset III	*M. mulatta*	11	606	617
Dataset IV	*M. musculus*	517	987	1,504

Supplementary Table S3: Datasets used as input in Experiment I

Dataset	Training	Test	Total
Positive (real)	334	83	417
Positive (synthetic) [1]	231	0	231
Negative (real)	565	142	707
Total (real) [2]	899	225	1124
Total (real + synthetic) [3]	1130	225	1355

[1] The synthetic samples were combined just in the training set.

[2] Dataset used as input in control groups

[3] Dataset used as input in experimental groups

Supplementary Table S4: Ratio real:synthetic for positive samples in Experiment II

	real:synthetic ratio					
Dataset	1:9	2:8	3:7	4:6	5:5	6:4[1]
Positive (real)	57	113	170	226	283	334
Positive (synthetic)	508	452	395	339	282	231
Negative (real) [2]	565	565	565	565	565	565
Total (real)	622	678	735	791	848	899
Total (real + synthetic)	1,130	1,130	1,130	1,130	1,130	1,130

[1] The 6:4 ratio is the same dataset as in Experiment I.

[2] The negative set is exactly the same negative set as in Experiment I.

Supplementary Table S5: Size of the datasets used as input in Experiment III

Dataset	Training	Test	Total
Positive (real)	173	43	216
Positive (synthetic) [1]	392	0	392
Negative (real)	565	142	707
Total (real) [2]	738	185	923
Total [3]	1,130	185	1,315

[1] The real and synthetic samples were combined just in training set.

[2] Dataset used as input in control groups

[3] Dataset used as input in experimental groups

3 Number of datasets in the experiments

Supplementary Table S6: Number of datasets created by approach in the Experiment I

Approach	Generated datasets	Balanced datasets
WGAN	10	10
FBGAN	10	10
SMOTE_A	10	10
SMOTE_B	10	10
SMOTE_C	10	10
Total	50	50

Table S6 shows the number of datasets created by each generative approach in the Experiment I. Next, each synthetic dataset was used once to balance a copy of the training set.

4 Number of misclassified samples

Figures S1 and S2 show the upset plots for misclassified samples by the seven tools in the Experiment I - FBGAN experimental group. Intersections are displayed as a matrix. Each row corresponds to one classification tool, and bar charts on the left show the number of misclassified by the tool. Each column corresponds to a possible intersection: the filled-in circles show which set is part of an intersection, and vertical bar charts show the size of the intersection.

Just one canonical miRNA was misclassified by the seven tools (Figure S1). miRNAClassification was the best tool (four misclassified sequences), while dnnPreMiR_CNN_RNN was the one that presented the most errors (11 misclassified sequences).

Two mirtrons were misclassified by the seven tools (Figure S2). HumanPreMiRNA_CNN was the best tool (two misclassified sequences), while dnnPreMiR_RNN was the one that presented the most errors (six misclassified sequences).

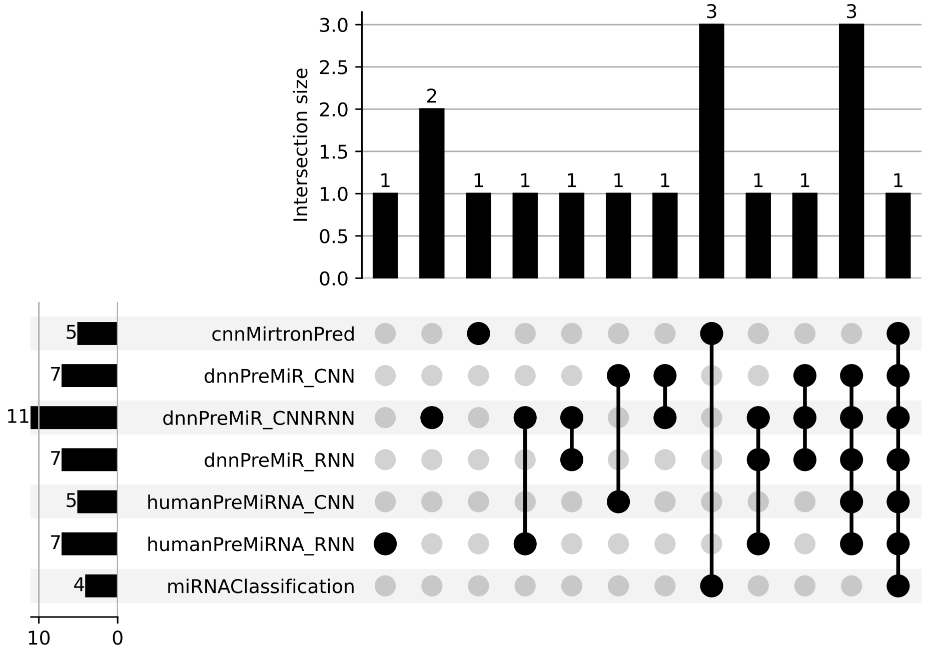

Supplementary Figure S1: **Upset plot misclassified canonical miRNAs**

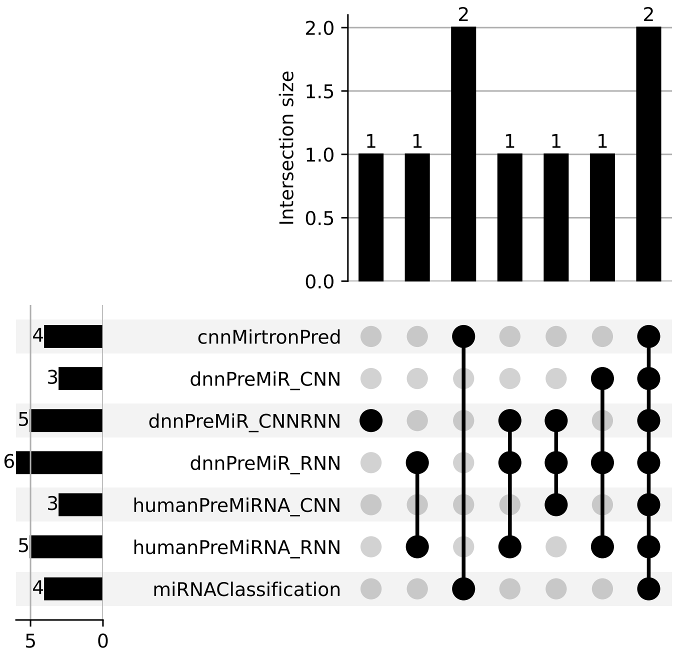

Supplementary Figure S2: **Upset plot misclassified mirtrons**

4.1 UMAP and PaCMAP analysis of training and test sets

Uniform Manifold Approximation and Projection (UMAP) (*5*) and Pairwise Controlled Manifold Approximation (PaCMAP) (*6*) were used to reduce the features of the samples to 2D vectors and visualize the data separation. The use of UMAP and PaCMAP complements the analysis perfomed with T-distributed Stochastic Neighbor Embedding (t-SNE).

A feature extraction step was employed to extract feature vectors based on Minimum Free Energy (MFE), sample length, GC content, and the count of nucleotides A, C, G, and T for each sample of the training and test sets. These features were passed as input into a UMAP (with default parameters) from the umap-learn python package (*5*) and PaCMAP (with default parameters) from the pacmap python package (*6*). The results are presented in Figures S3, S5 and S6.

Figure S3 shows the UMAP projections of the training set of Control Group and FBGAN experimental groups. The synthetic mirtron samples generated by FBGAN significantly overlap with the real mirtron samples (Fig. S3A). On the other hand, the synthetic samples generated by SMOTEA do not exhibit this same overlap, despite being concentrated at the real mirtron samples (Fig. S3B).

Figure S4 shows the UMAP projections of the test set. Larger points represent the 56 incorrectly classified samples in the control group (Fig. S4A) and the 26 incorrectly classified samples in the experimental FBGAN group (Fig. S4B). It can be observed that misclassified samples are predominantly located in regions with intersection between the two classes of samples. However, when compared to the experimental FBGAN group, it is evident that there is a significant reduction in the number of incorrectly classified samples, especially in the intersection region of the data. These misclassifications may be due to the inherent overlap in the feature space between different classes, making it difficult for the model to distinguish them in such regions. The same observation can be made when using PaCMAP (Figures S5 and S6).

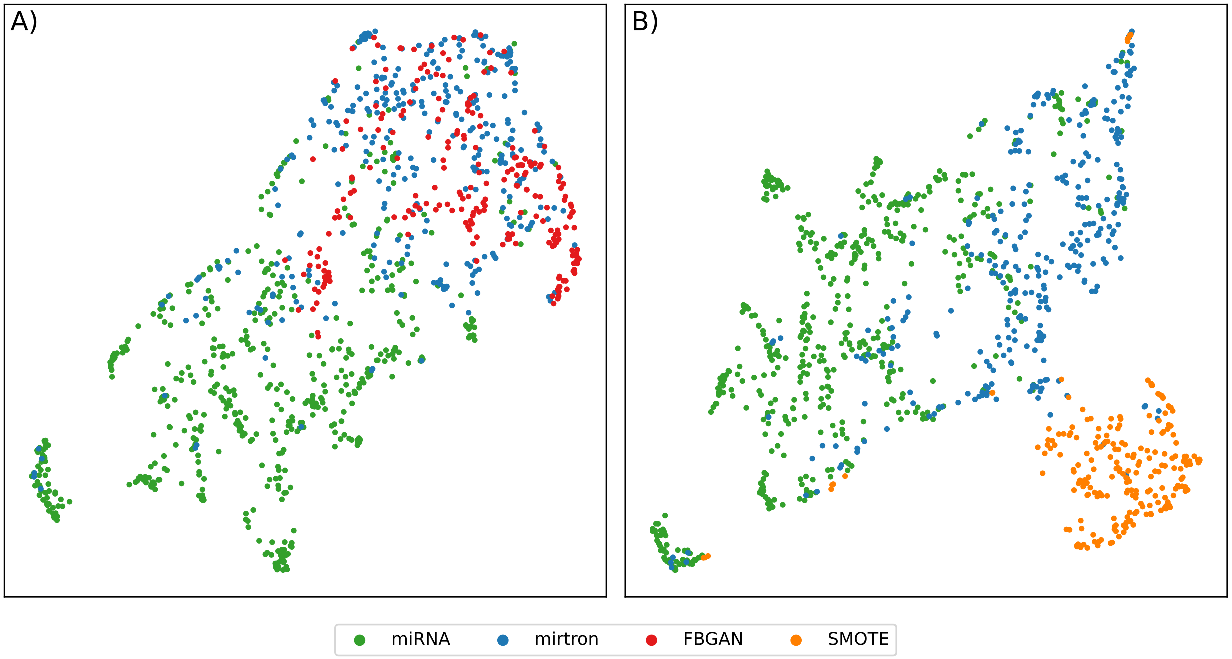

Supplementary Figure S3: **UMAP analysis of training sets for FBGAN and SMOTEA experimental groups.** The **A** plot shows the distribution of the FBGAN experimental group, while the **B** plot shows the SMOTEA group. The miRNA and mirtron categories are represented by green and blue points, respectively, and the synthetic data points are represented in red (**A**) and orange (**B**).

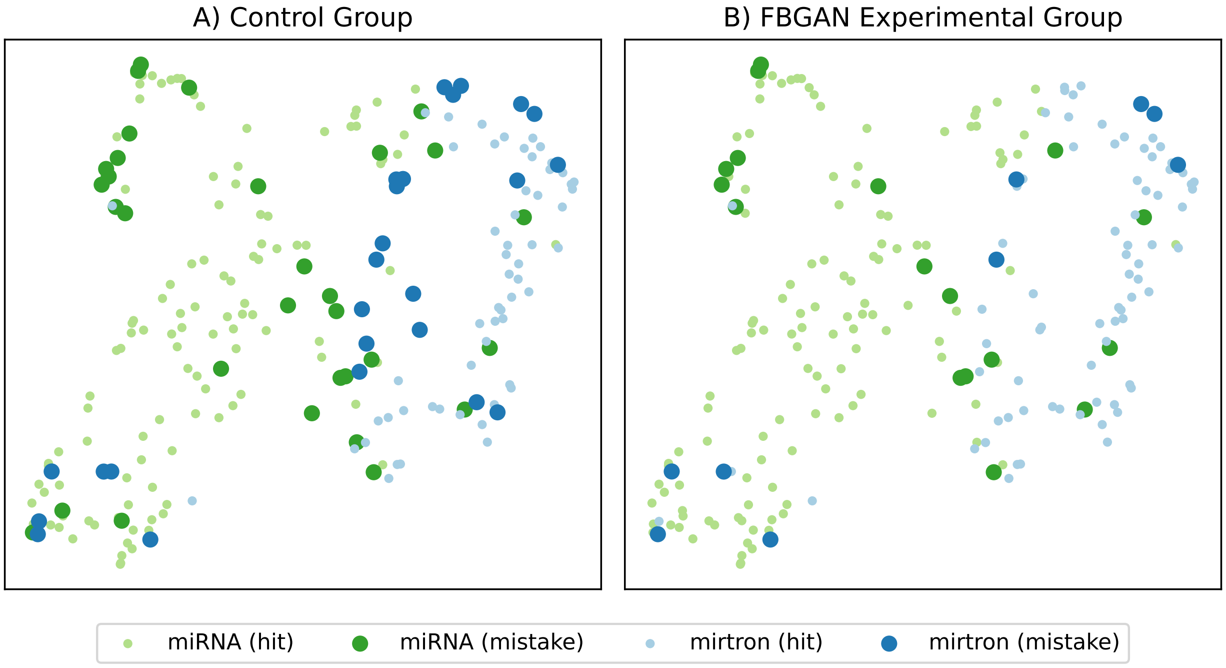

Supplementary Figure S4: **UMAP analysis of test set and the classification results of all tools.** Both the plots show the two-dimensional space of test set calculated by UMAP. The green and blue points represent the miRNA and mirtrons data, and the big points represent the misclassified samples by the tools. The **A** plot represents the results based on control group, while the **B** plot shows the results based on FBGAN experimental group.

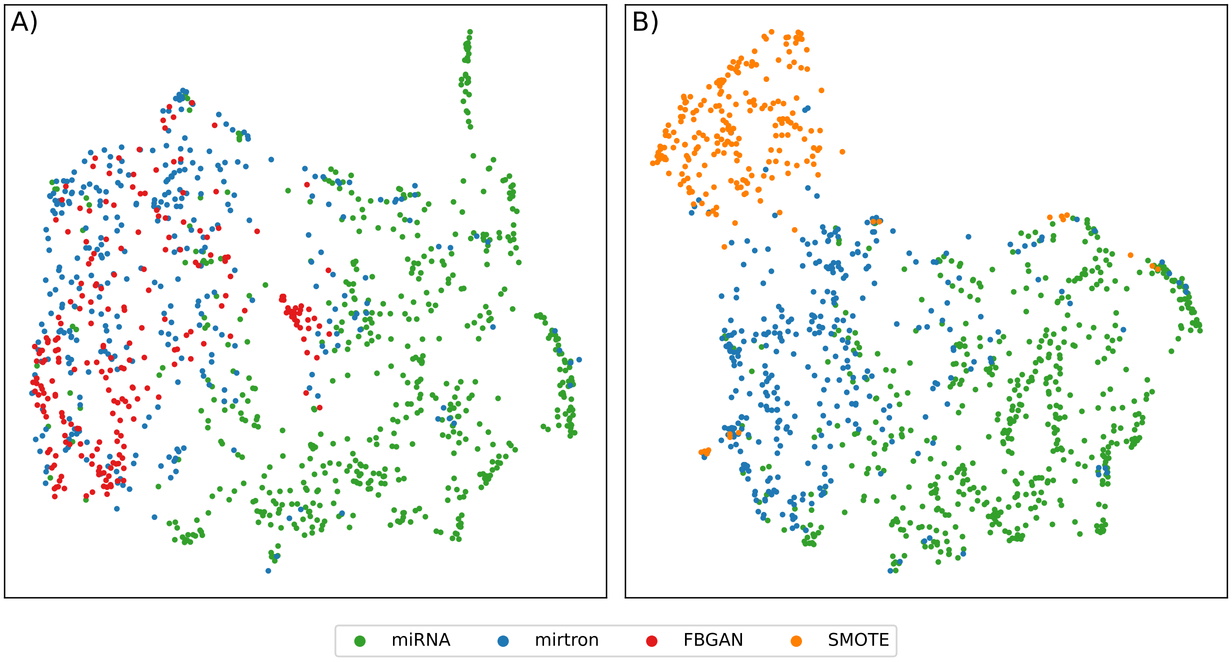

Supplementary Figure S5: **PaCMAP analysis of training sets for FBGAN and SMOTEA experimental groups.** The **A** plot shows the distribution of the FBGAN experimental group, while the **B** plot shows the SMOTEA group. The miRNA and mirtron categories are represented by green and blue points, respectively, and the synthetic data points are represented in red (**A**) and orange (**B**).

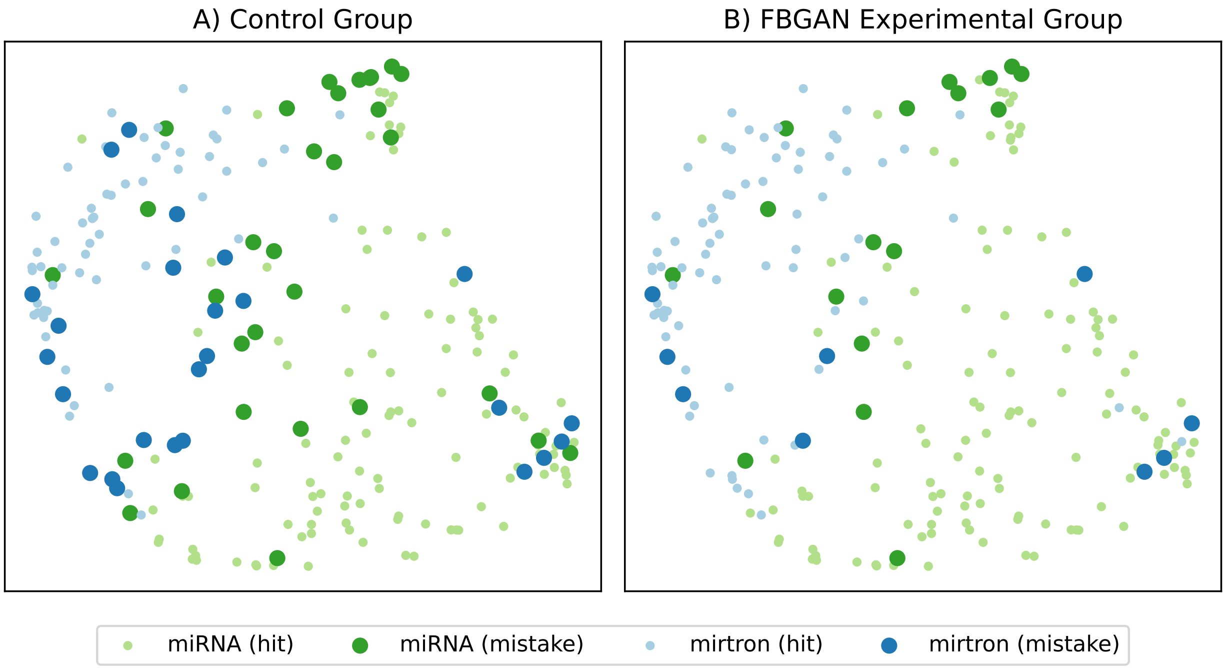

Supplementary Figure S6: **PaCMAP analysis of test set and the classification results of all tools.** Both the plots show the two-dimensional space of test set calculated by PaCMAP. The green and blue points represent the miRNA and mirtrons data, and the big points represent the misclassified samples by the tools. The **A** plot represents the results based on control group, while the **B** plot shows the results based on FBGAN experimental group.

5 Details of Experiment I

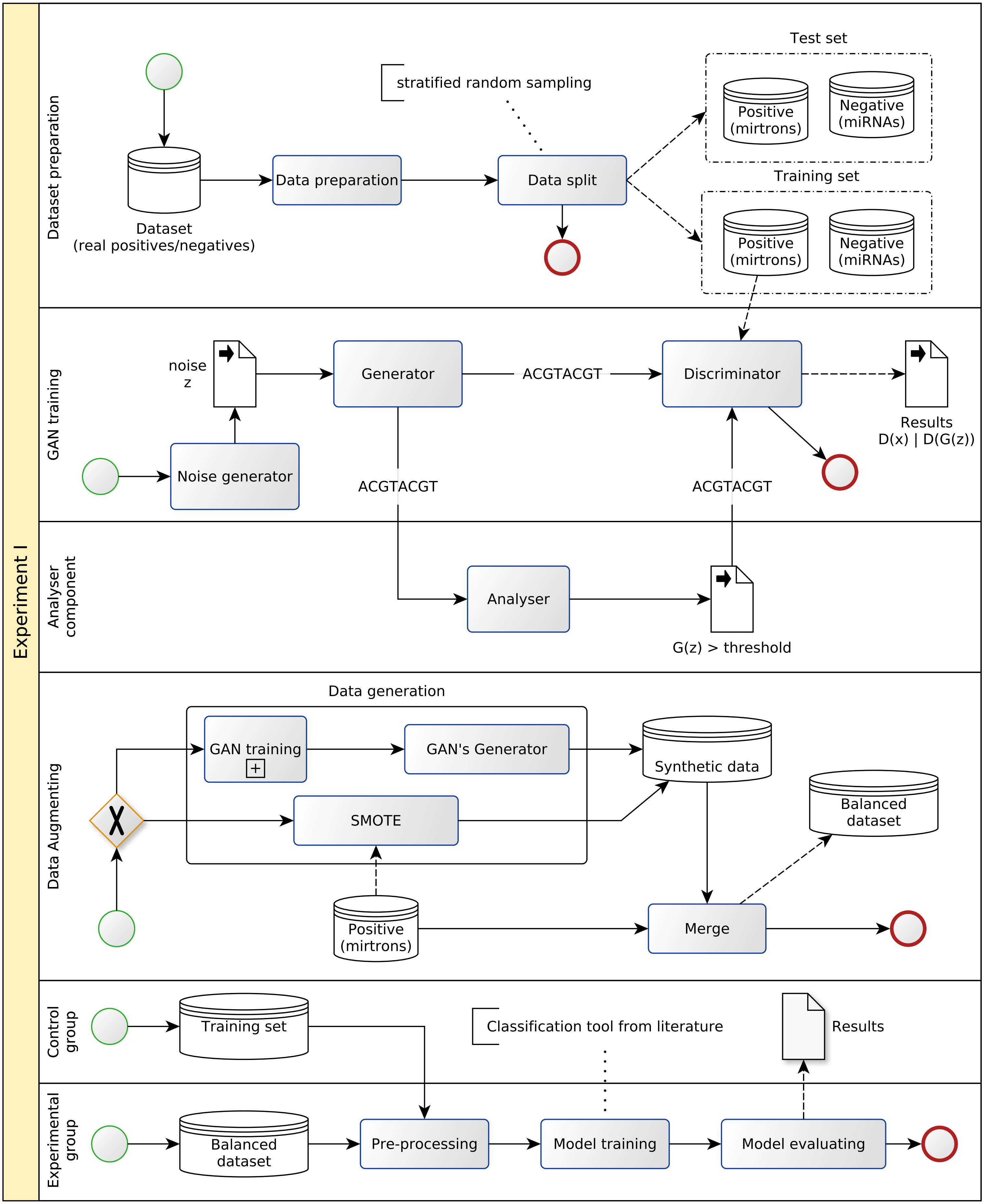

Supplementary Figure S7: **Business Process Model and Notation (BPMN) (*7*) representation for Experiment I.** The methodology of preparing the dataset, training a GAN, generating synthetic data to apply data augmentation and balancing the training set, and the process to evaluate the GANs (obtaining the results of the control and experimental groups) are represented in process format.

6 Classification performance on other species (Experiment IV)

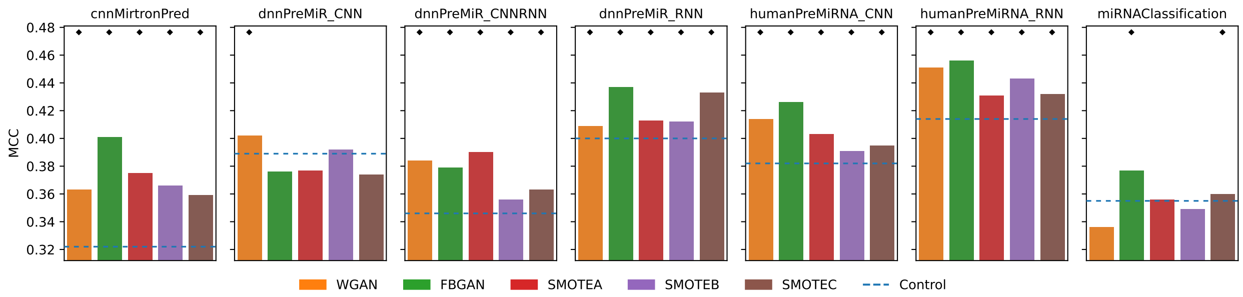

Supplementary Figure S8: **Results for Experiment IV using Dataset II (*H. sapiens*).** Each subplot corresponds to a different classification tool, and the colored bars represent the MCC of the classification by the corresponding model of the experimental groups. The blue horizontal line indicates the MCC of the control group for each classification tool. The values above the bars show the improvement in MCC compared to the control. A diamond character (◆) above a bar denotes an improvement $\geq 1\%$.

For the Dataset II (*H. sapiens*), 12 out of 14 ($\approx 85.7\%$) models trained with GAN data showed superior results compared to the control group model (Figure S8). Of the models trained with SMOTE, 16 out of 21 ($\approx 76.2\%$) showed improvement. Only the dnnPreMiR_CNN and miRNA-Classification tools had results below the control group. When trained with synthetic data, the cnnMirtronPred models achieved the highest MCC increase.

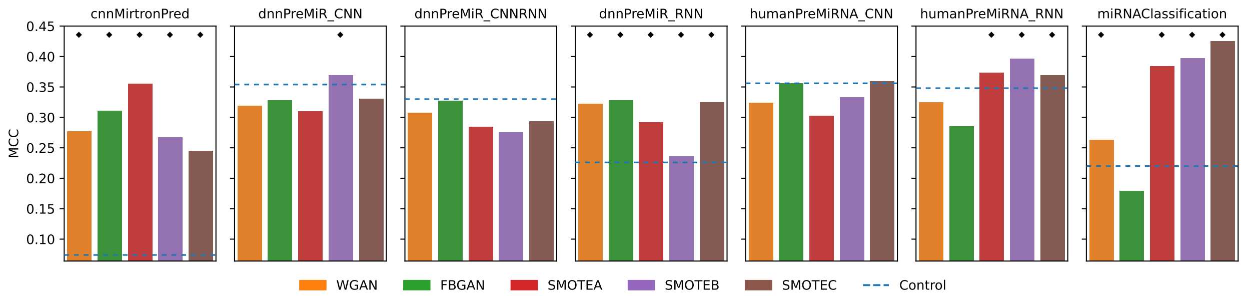

Supplementary Figure S9: **Results for Experiment IV using Dataset III (*M. mulatta*).** Each subplot corresponds to a different classification tool, and the colored bars represent the MCC of the classification by the corresponding model of the experimental groups. The blue horizontal line indicates the MCC of the control group for each classification tool. The values above the bars show the improvement in MCC compared to the control. A diamond character (◆) above a bar denotes an improvement $\geq 1\%$.

The classification of Dataset III (*Macaca mulatta* samples) (Figure S9) by the cnnMirtronPred and miRNAClassification tools showed distinct results compared to the other tools. Models of these two tools showed an improvement in generalization ability (except for the miRNAClassification model trained with FBGAN synthetic data). In contrast, the other tools showed little or no improvement in generalization when using synthetic data for model training. Once again, cnnMirtronPred had the highest increase in generalization ability.

7 GAN Components

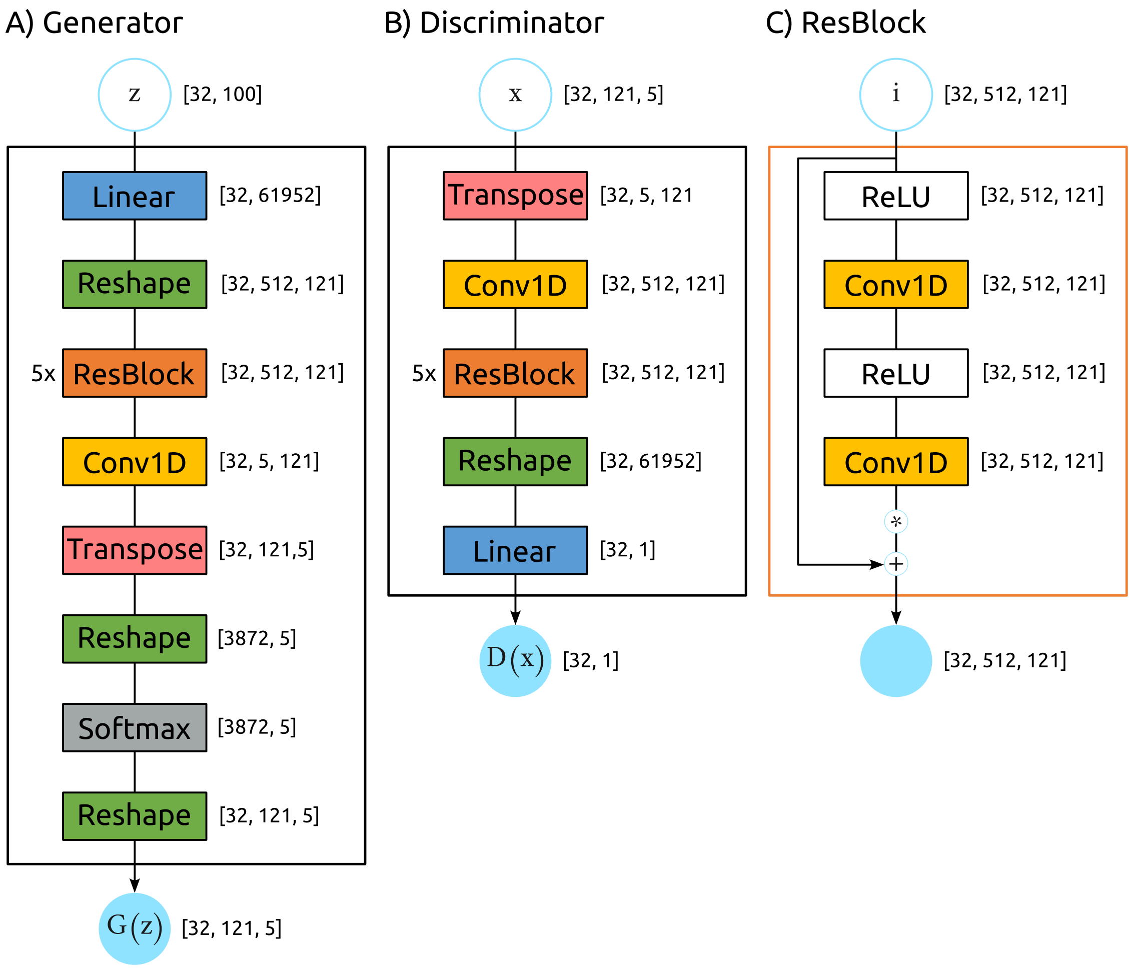

Supplementary Figure S10: **Components of WGAN-GP and FBGAN of this work**: **(A)** Generator; **(B)** Discriminator and **(C)** ResBlock used by Discriminator and Generator. The rectangles represent the layers of the components. The input and output data are represented by unfilled and filled circles, respectively. The number to the right of each rectangle/circle indicates the dimension of the resulting matrix.

Supplementary Table S7: Discriminator architecture

	Layer	Output shape	Output shape (applied values)
	Input	[batch_size, seq_len, n_chars]	[32, 121, 5]
1	Transpose	[batch_size, n_chars, seq_len]	[32, 5, 121]
2	Conv1D[1]	[batch_size, hidden_layers, seq_len]	[32, 512, 121]
3	5×ResBlock [2]	[batch_size, hidden_layers, seq_len]	[32, 512, 121]
4	Reshape	[batch_size, hidden_layers * seq_len]	[32, 61952]
5	Linear	[batch_size, 1]	[32, 1]

[1] in=n_chars, out=hidden_layers, kernel=1

[2] dim_size=hidden_layers

Supplementary Table S8: Generator architecture

	Layer	Output shape	Output shape (applied values)
	Input	[batch_size, 100]	[32, 100]
1	Linear	[batch_size, hidden_layers * seq_len]	[32, 61952]
2	Reshape	[batch_size, hidden_layers, seq_len]	[32, 512, 121]
3	5×ResBlock [1]	[batch_size, hidden_layers, seq_len]	[32, 512, 121]
4	Conv1D	[batch_size, n_chars, seq_len]	[32, 5, 121]
5	Transpose	[batch_size, seq_len, n_chars]	[32, 121, 5]
6	Reshape	[batch_size * seq_len, n_chars]	[3872, 5]
7	Softmax [2]	[batch_size * seq_len, n_chars]	[3872, 5]
8	Reshape	[batch_size, seq_len, n_chars]	[32, 121, 5]

[1] dim_size=hidden_layers

[2] gumbel_softmax($\tau = 0.5$)

Supplementary Table S9: ResBlock(dim_size) architecture

	Layer	Output shape	Output shape (applied values)
	Input	[batch_size, dim_size, seq_len]	[32, 512, 121]
1	ReLU	[batch_size, dim_size, seq_len]	[32, 512, 121]
2	Conv1d [1]	[batch_size, dim_size, seq_len]	[32, 512, 121]
3	ReLU	[batch_size, dim_size, seq_len]	[32, 512, 121]
4	Conv1d [1]	[batch_size, dim_size, seq_len]	[32, 512, 121]

[1] in=dim_size, out=dim_size, kernel=n_chars, padding=2

Supplementary Table S10: Hyperparameters used in the training of the GANs

Var	Value	Description
batch_size	32	
seq_len	121	The max Sequence Length
n_chars	5	[A,C,G,T,P]
hidden_layers	512	Number of hidden layers

Supplementary Tables S7, S8, and S9 present the layer structures of the Discriminator, Generator, and ResBlock components, respectively. The first column (#) indicates the layer's position within the architecture, while the second column (Layer) specifies the type of layer used. The third column (Output Shape) describes the dimensions of the output matrix, clarifying the relationship between the model parameters. Finally, the last column provides the parameter values used during GAN training. Supplementary Tables S10 present the hyperparameters used during the training of the GANs.

8 Calculating the improvement of the experimental group

$$\frac{\sum_i(\sum_{it} MCC - \sum_{ct} MCC)}{7} \tag{S1}$$

The Equation S1 represents the sum of differences in MCC values between experimental and control groups across various tools. Specifically: i denotes an experimental group; c represents the corresponding control group for a given tool; t refers to a specific tool; and 7 is the number of classification tools used (Table S1).

This formulation calculates the net difference in MCC values between experimental and control groups across all tools. For example, to calculate the improvement achieved by the i = FBGAN experimental group, we applied Equation S1. Initially, we considered t = cnnMirtronPred for simplification purposes.

$$\begin{aligned}\sum_{ct} MCC &= 0.817 + 0.799 + 0.827 + 0.828 + 0.818 \\ &+ 0.810 + 0.777 + 0.818 + 0.822 + 0.808 \\ &= 8.124\end{aligned} \tag{S2}$$

$$\begin{aligned}\sum_{it} MCC &= 0.876 + 0.834 + 0.876 + 0.876 + 0.860 \\ &+ 0.876 + 0.877 + 0.861 + 0.860 + 0.833 \\ &= 8.629\end{aligned} \tag{S3}$$

$$\sum_{it} MCC - \sum_{ct} MCC = 8.629 - 8.124 = 0.505 \tag{S4}$$

By calculating $\sum_{it} MCC$ and $\sum_{ct} MCC$ for all seven tools, we obtain:

$$\begin{aligned}\frac{\sum_i}{7} &= \frac{0.505 + 0.506 + 0.605 + 0.474 + 0.662 + 0.628 + 0.466}{7} \\ &= \frac{3.846}{7} = 0.549\end{aligned} \tag{S5}$$

References and Notes

1. Zheng, X., Xu, S., Zhang, Y. & Huang, X. Nucleotide-level Convolutional Neural Networks for Pre-miRNA Classification. *Scientific Reports* **9**, 628 (2019).

2. Zheng, X., Fu, X., Wang, K. & Wang, M. Deep neural networks for human microRNA precursor detection. *BMC Bioinformatics* **21**, 17 (2020).

3. Rezoun, A. S. M. *et al.* Supervised Deep Learning Methods for Human pre-miRNA Identification. In *2020 IEEE Region 10 Symposium (TENSYMP)*, 1098–1101 (IEEE, Dhaka, Bangladesh, 2020).

4. Zahid Bin Aziz, A. & Al Mehedi Hasan, M. A Mixed Convolutional Neural Network for Pre-miRNA Classification. In *3rd International Conference on Electrical, Computer and Telecommunication Engineering, ICECTE 2019*, 217–220 (2019).

5. McInnes, L., Healy, J. & Melville, J. UMAP: Uniform manifold approximation and projection for dimension reduction (2018). `1802.03426`.

6. Wang, Y., Huang, H., Rudin, C. & Shaposhnik, Y. Understanding how dimension reduction tools work: An empirical approach to deciphering t-SNE, UMAP, TriMap, and PaCMAP for data visualization. *Journal of Machine Learning Research* **22**, 1–73 (2021).

7. BPMN Specification - Business Process Model and Notation. https://www.bpmn.org/.
